# Increased quality of *in natura* and cryopreserved semen of water buffaloes supplemented with saturated and unsaturated fatty acids from the palm oil industry

**DOI:** 10.1590/1984-3143-AR2020-0522

**Published:** 2020-11-24

**Authors:** Lílian Kátia Ximenes Silva, José de Brito Lourenço, Aluizio Otavio Almeida da Silva, José Silva de Sousa, André Guimarães Maciel e Silva, Adriana Novaes dos Reis, Moysés dos Santos Miranda, Simone do Socorro Damasceno Santos, Otávio Mitio Ohashi, Lucieta Guerreiro Martorano, Geraldo Narciso da Rocha, Cristian Faturi, Eziquiel de Morais, Érica Karine Lourenço Mares, Alexandre Rossetto Garcia

**Affiliations:** 1 Instituto de Medicina Veterinária, Universidade Federal do Pará, Castanhal, PA, Brasil; 2 Centro de Biotecnologia em Reprodução Animal, Universidade Federal do Pará, Castanhal, PA, Brasil; 3 Laboratório de Fertilização in Vitro, Instituto de Ciências Biológicas, Universidade Federal do Pará, Belém, PA, Brasil; 4 Empresa Brasileira de Pesquisa Agropecuária – Embrapa Amazônia Oriental, Belém, PA, Brasil; 5 Faculdade de Química, Universidade Federal do Pará, Belém, PA, Brasil; 6 Instituto de Saúde e Produção Animal, Universidade Federal Rural da Amazônia, Belém, PA, Brasil; 7 Empresa Brasileira de Pesquisa Agropecuária – Embrapa Pecuária Sudeste, São Carlos, SP, Brasil

**Keywords:** spermatozoa, cryopreservation, sperm membranes, lipid profile, diet

## Abstract

Ruminant energy supplementation with vegetable oils or fats has been standing out worldwide and oil palm processing has been receiving growing interest. This study assessed the effect of supplementation with saturated and unsaturated fatty acids from the palm oil industry on the lipid profile of seminal plasma and of the sperm membrane, as well as on the morphological and functional characteristics of raw and cryopreserved buffalo semen. Twelve purebred Murrah bulls (*Bubalus bubalis*) were assigned to the experimental groups and fed diets for 120 days with no added lipids (CONT, four bulls), or with an extra amount of 3% lipids from crude palm oil (PALM, four bulls), or from palm oil deodorizer distillate (PODD, four bulls). Semen was collected and cryopreserved every 15 days. The lipid composition of membranes and semen quality were determined after collections. Lipid supplementation did not impact feed intake (P>0.05). Diet enrichment with PALM increased the linoleic acid (C18:2,ω6) in seminal plasma. Lipid supplementation did not increase the polyunsaturated fatty acids in the sperm membrane composition, but significantly increased the lignoceric acid (C24:0). Cryopreserved semen of the supplemented bulls presented higher progressive motility (60.2 vs. 67.9 vs. 65.2%; P<0.05) and sperm viability detected by eosin-nigrosin staining (61.1 vs. 69.4 vs. 67.8%; P<0.05). Palm oil reduced major sperm defects in both raw (12.2 vs. 9.3 vs. 13.2%; P<0.0001) and cryopreserved semen (12.4 vs. 9.4 vs. 11.2%; P<0.0001). The lipids added to the diet did not impact the population of spermatozoa with intact plasma and acrosomal membranes (PI-/PSA-), but significantly increased the percentage of spermatozoa with high mitochondrial potential (25.6 vs. 31.5 vs. 32.0%; P=0.008). The results suggest that lipid supplementation based on crude palm oil or palm oil deodorizer distillate can be safely used to feed buffalo bulls and may increase sperm attributes related to male fertility.

## Introduction

The characteristics that provide better quality to semen may be directly influenced by nutritional factors (Barth et al., 2008). Among those, dietary lipid composition has shown positive effects on semen quality in different animal species (Mitre et al., 2004; Roqueta-Rivera et al., 2010). Dietary lipid profiles may impact the proportion of phospholipids in sperm membranes by changing the fertilizing capacity of spermatozoa (Santos et al., 2014; Van Tran et al., 2017). Thus, fatty acid composition in spermatozoa has been considered an important factor for male fertility (Safarinejad et al., 2010; Nasrallah et al., 2020).

Among the polyunsaturated fatty acids found in water buffalo sperm, linolenic acid (C18:3, ω3) and linoleic acid (C18:2, ω6) stand out (Jain and Anand, 1976). Those acids are considered essential as they are not synthesized in the organism of the animals and must be provided in the diet (Perini et al., 2010). Relatively high proportions of polyunsaturated fatty acids in spermatozoa enhance sperm capacitation and acrosomal reaction (Lenzi et al., 2000; Furland et al., 2007). In addition, fatty acid composition in spermatozoa has proven to be important for the cryopreservation of those cells in some animal species (Maldjian et al., 2005; Melville et al., 2012; Lee et al., 2019).

Sperm cryopreservation aims to preserve cells below the freezing point of water and to maintain cell composition and viability indefinitely (Pegg, 2002). However, this biotechnique causes irreversible damages to the plasma membrane, to the acrosome, and to the mitochondria of spermatozoa (Minervini et al., 2013; Kumar et al., 2014), which, consequently, reduces semen quality after thawing (Maldjian et al., 2005; Samadian et al., 2010). It is known that cryopreservation exerts detrimental effects inducing cryocapacitation damages especially in buffalo semen (Longobardi et al., 2017). That includes a decrease in sperm viability and motility, more evident in buffalo compared to cattle (Andrabi, 2009). During cryopreservation, the proportion of polyunsaturated fatty acids in spermatozoa decreases (Cerolini et al., 2006; Ejaz et al., 2014; Khoshvaght et al., 2016) and lipids are redistributed in the sperm membranes. Changes in lipid orientation destabilize sperm membrane, which increases its permeability and may lead to rupture (Silva and Gadella, 2006; Silva et al., 2009). Therefore, it is assumed that modifying semen fatty acid profile through lipid supplementation could increase spermatozoa resistance and protect them from cryopreservation damage (Celeghini et al., 2008).

Recent researches on the adoption of vegetable oils or fats as alternative energy sources for ruminants have been carried out (Esmaeili et al., 2014; Gürler et al., 2015; Moallem et al., 2015; Khoshvaght et al., 2016), which has brought attention to the inclusion of agro-industry products in ruminant feed (Van Tran et al., 2017). Among such products are by-products from oil palm (*Elaeis guineensis*) fruit processing (Santos et al., 2014; Gonçalves et al., 2014; Silva et al., 2014), which are available in many parts of the world. During industrialization, palm oil is extracted from the pulp and its physical refining generates a compound known as palm oil deodorizer distillate. Both compounds are rich in saturated and unsaturated fatty acids at different proportions (Grupo Agropalma, 2010).

Adding saturated and unsaturated fatty acids from alternative sources could stimulate the use of agro-industrial products and by-products in buffalo feed. If they were shown to be effective in increasing semen quality, lipid sources from the palm oil industry could be employed to feed buffalos used in natural mount or kept in artificial insemination centers. Thus, this study aimed to assess the effect of dietary supplementation of buffalo bulls with crude palm oil and with its deodorizer distillate, rich sources in saturated and unsaturated fatty acids, on the lipid composition of semen (seminal plasma and sperm membrane) and on morphological and functional characteristics of spermatozoa before and after cryopreservation.

## Methods

### Site and period

The trial was conducted at the Center of Biotechnology of Animal Reproduction, an approved artificial insemination center of Federal University of Pará (CEBRAN/UFPA), Castanhal, PA, Brazil (1°18’S, 47°56’W). The site has megathermal humid tropical climate (Afi according to Köppen) with annual rainfall between 2300 and 2800 mm and monthly rainfall from 67 to 399 mm (Alvares et al., 2013). The experiment was carried out from November 2014 to December 2015, and comprised the less rainy season of the year, when buffaloes are sexually active in Brazilian Amazon (Garcia, 2006).

All experimental procedures were approved by the Ethics Commission on Animal Use (CEUA) under protocol 02-15. The results were reported according to The Animals in Research: Reporting *in Vivo* Experiments Guidelines - ARRIVE (Kilkenny et al., 2011).

### Animals

Twelve water buffalo (*Bubalus bubalis*) at 50.4±8.6 months old, 673.6±94.0 kg, that were part of a commercial batch of Murrah purebred bulls were used as semen donors. The animals had previously known semen profiles (Table 1) and were homogeneously assigned to groups Control (CONT, four bulls), Crude Palm Oil (PALM, four bulls), and Palm Oil Deodorizer Distillate (PODD, four bulls). The animals were individually housed in adjacent pens under the same environmental conditions and sanitary management and were submitted to the same semen collection regimen. One animal from the PODD group was removed after five weeks due to a health issue unrelated to the study and its samples were not included in the results.

**Table 1:** Data on sperm characteristics (mean±standard deviation) of buffalo bulls at the beginning of the experiment.

**Variable**	**CONT (4 bulls)**	**PALM (4 bulls)**	**PODD** **^†^** **(4 bulls)**
Progressive Motility (%)	70.0±4.1	70.0±3.5	66.7±2.4
Vigor (%)	2.7±0.6	2.5±0.7	3.0±0.0
Viability (%)	67.3±4.5	70.5±3.5	58.0±15.1
Major Defects (%)	7.3±1.5	10.5±2.1	13.3±9.7
Minor Defects (%)	7.3±1.5	8.0±1.4	2.7±16.0
Total Defects (%)	14.7±7.2	18.5±3.5	16.0±11.5

† CONT = control group; PALM = crude palm oil group; PODD = palm oil deodorization distillate group.

### Experimental design

**Figure 1:**
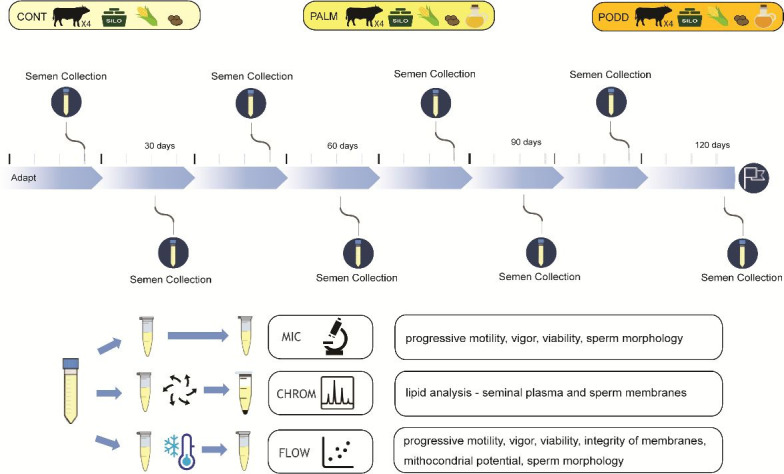
Experimental design with indication of the dietary treatments adopted over 120 days, number of bulls per treatment, semen collection frequency, sample processing, and analyses carried out.

The study used a completely randomized design with three treatments (diets), four repetitions (animals) per treatment, and repeated measures over time (eight semen collections per animal). Previously to the experiment, bulls had been routinely submitted to 2-3 semen collections/week as usually adopted at CEBRAN artificial insemination center. Thus, at the beginning of the experiment, the bulls were reproductively active and presented semen parameters within the expected range for the species (CBRA, 2013). The animals were fed experimental diets for 120 days, of which the first 15 were used for adaptation to the nutritional management. After the adaptation, the animals underwent semen collection every 15 days for a total of eight ejaculates collected per bull (88 ejaculates). After collection, the semen was divided into three aliquots. One aliquot was immediately used for semen quality assessment, another one was used to obtain the seminal plasma and spermatozoa fractions for lipid profile analysis, and the third one was cryopreserved and later assessed for semen quality after thawing (Figure 1).

### Diets

The experimental diets were composed of elephant grass (*Pennisetum purpureum*) silage and concentrate based on ground corn kernels (*Zea mays*) and whole beans (*Phaseolus vulgaris*) to be isoproteic. The control diet received no lipid supplementation while the others were added with 3% crude palm oil or 3% palm oil deodorization distillate (Table 2). The diets were provided twice a day (8 A.M. and 4 P.M.) in individual feeding troughs allowing for 10% leftovers. Every morning, the leftovers from the previous day were weighed to calculate daily intake. The animals had *ad libitum* access to mineral mix in a covered trough and to water in an automated drinking trough.

**Table 2:** Diet ingredients and chemical composition of the concentrates provided to buffalo bulls in semen collection regimen according to the different treatments.

**Ingredients**	**CONT**	**PALM**	**PODD** **^†^**
Ground corn kernels (% DM)^‡^	23.22	13.86	13.86
Ground beans (% DM)	10.00	10.00	10.00
Crude palm oil (% DM)	-	3.00	-
Palm oil deodorization distillate (% DM)	-	-	3.00
Urea (% DM)	1.05	1.26	1.26
Elephant grass silage (% DM)	65.73	71.88	71.88
Chemical composition			
Dry matter (%)	88.21	91.78	89.35
Crude protein (% DM)	20.19	21.15	21.02
Ether extract (% DM)	3.69	16.17	15.32
Neutral detergent fiber (% DM)	25.61	19.52	25.70
Neutral detergent fiber (% DM)	5.03	3.57	4.56

† CONT = control group; PALM = crude palm oil group; PODD = palm oil deodorization distillate group; ‡ DM = dry matter.

The fatty acid composition of the lipid sources used in the experiment was previously analyzed by gas chromatography (Table 3).

**Table 3:** Fatty acid composition (%) of crude palm oil (PALM) and of palm oil deodorization distillate (PODD) used in the dietary supplementation of buffalo bulls.

**Fatty acid (%)**	**PALM**	**PODD**
Lauric acid (C12:0)	0.19	0.25
Myristic acid (C14:0)	0.59	0.72
Palmitic acid (C16:0)	37.97	46.05
Stearic acid (C18:0)	4.61	4.74
Arachidic acid (C20:0)	0.33	0.29
Palmitoleic acid (C16:1)	0.11	-
Oleic acid (C18:1)	46.12	39.06
Linoleic acid (C18:2)	9.52	8.37
Linolenic acid (C18:3)	0.24	0.22
Σ SFA ^†^	43.69	52.05
Σ UFA ^‡^	55.99	47.65
UFA/SFA^§^	1.28	0.91

† Σ SFA, sum of saturated fatty acids; ‡ Σ UFA, sum of unsaturated fatty acids; § UFA/SFA, ratio between unsaturated and saturated fatty acid contents.

### Semen collection and assessment of lipid composition in the seminal plasma and sperm membrane

The semen was collected using an artificial vagina (Vale et al., 1991). The semen was kept at 37 °C and divided into three aliquots. One aliquot of *in natura* semen was centrifuged at 600 *g* for 10 min to obtain a pellet containing the spermatozoa and the supernatant containing the seminal plasma. An amount of 4 µL of both samples were used for lipid extractions from the sperm membrane and seminal plasma, respectively (Bligh and Dyer, 1959). The lipid profile of fatty acid methyl esters (FAMEs) was determined through gas chromatography using the same protocol for seminal plasma and sperm cells. FAMEs were prepared using AOCS Ce 2-66 method. In a typical procedure, 200 mg of the sample was weighed and transferred to a 50 mL round-bottomed flask. Then 4 mL of the 0.5 mol/L NaOH methyl solution was added to the flask and the system was maintained under constant stirring and reflux (60 °C). After 10 min, 5 mL of BF3 solution (14% in methanol) was added to the flask and the system was kept under constant stirring and reflux, in order to heat the solution in a controlled manner at a constant temperature (60 °C). After the mentioned time, 5 mL of heptane was added to the flask. After that, 15 mL of saturated NaCl solution was added to the flask. The system was kept at rest for 10 min and after separation of the phases, 1 mL of supernatant was collected for later analysis in gas chromatography. Analysis by chromatography was performed by the AOCS Ce 1a-13 method using a chromatograph equipped with an automatic injector (CP 3800 Varian, Walnut Creek, CA, USA) and a flame ionization detector. The chromatograph had a CP WAX 52 CB capillary column with 3 m length x 0.32 mm inner diameter and 0.25 μm film and used helium as carrier gas at a flow rate of 1.0 mL/min. For the analyses, 1 μL of the esterified sample was injected into the chromatograph. Seven fatty acid (methyl ester) peaks were identified by comparing the retention time with standard FAME blends (Rodrigues et al., 2010).

### Raw semen evaluation

Semen samples were maintained at 37 °C during the execution of the analyses. Visual progressive motility (%) and sperm vigor (1 to 5 scale) analyses were performed on a prewarmed slide using a phase-contrast microscope (200 X) in accordance with Ejaz et al. (2017). Spermatozoa viability (%) was evaluated with eosin-nigrosin supravital staining. For this, 10 μL of raw semen was placed on a prewarmed slide and mixed with 10 μL of stain (1% eosin and 5% nigrosin in 3% trisodium). Differential evaluation was carried out on 200 cells per sample using bright-field microscopy (1,000 X). The spermatozoa whose heads were stained in pink or partially stained were classified as dead while non-stained ones were considered alive and presenting structural integrity of the plasma membrane (Iqbal et al., 2010; Khoshniat et al., 2020). Semen samples were always evaluated by the same experienced technician.

### Morphological abnormalities

The sperm morphology analysis was performed under phase-contrast microscopy, with a magnification of 1,000 X. Semen samples were prediluted in buffered formalin solution (1:25) and 200 cells per sample were evaluated. The abnormal cells were classified according to their morphological characteristics, as carriers of minor defects (%) or major defects (%). The total defects (%) represent the sum of both categories in each sample (Blom, 1973). Semen samples were always evaluated by the same experienced technician.

### Semen cryopreservation

For cryopreservation, the semen was diluted with no removal of seminal plasma in a TES-TRIS based medium (4.9% TES, 1.06% TRIS, 11% skimmed milk, 20% egg yolk, 7% glycerol, 0.21% fructose, 1000 UI/mL penicillin and 1 g/mL streptomycin) to reach a total sperm number of 10 million per dose or a final concentration of 40x10^6^ spermatozoa/mL. The semen was placed in 0.25 mL French straws that were cooled and remained in equilibrium for 3 hours at 5 °C. Subsequently, it was submitted to liquid nitrogen vapor for 20 minutes, and then plunged into it (-196°C) as described by Vale et al. (1991). The straws were stored in liquid nitrogen for at least 30 days prior to analyses.

### Post-thawing semen evaluation and flow cytometry

The cryopreserved semen was thawed at 35 °C for 30 s. The samples were analyzed for progressive motility, vigor, and sperm morphology as previously described. Sperm membrane and acrosome integrity was assessed by flow cytometry (BD FACSCanto II, Franklin Lakes, NJ, USA) using the technique of associating propidium iodide (PI; Sigma, 28,707-5, Saint Louis, MO, USA) and fluorescein isothiocyanate-*Pisum sativum* agglutinin (FITC-PSA; Sigma, L-0770, Saint Louis, MO, USA). Mitochondrial potential was assessed in a flow cytometer using a 5,5’,6,6’-Tetrachloro-1,1’3,3’-tetraethylbenzimidazolocarbocyanine iodide probe (JC-1; Sigma, T-4069, Saint Louis, MO, USA) (Celeghini et al., 2010).

For flow cytometry, two straws of each ejaculate were thawed and their contents were joined in a single microtube. The thawed semen was separated by Percoll gradient (90 to 45%) and then washed and centrifuged in TALP medium. Next, a 10 µL semen sample was added with 3 µL PI (2 µg/mL) and 30 µL FITC-PSA (100 µg/mL). Another 10 µL sample was added with 2 µL JC-1 (153 µM) (Celeghini et al., 2010). The samples were incubated at 38.5 °C for 10 min and then submitted to flow cytometry. In each sample, 10,000 spermatozoa were analyzed. The positive controls used in the analyses were semen samples that, after separation in Percoll, were flash frozen with no added cryoprotectant to result in a large proportion of sperm cells with damaged membranes (Celeghini et al., 2007; Santana et al., 2016).

The evaluation with conjugated probes allowed classifying the spermatozoa as cells with intact plasma membrane and acrosome (PI-/PSA-), intact plasma membrane and injured acrosome (PI-/PSA+), injured plasma membrane and intact acrosome (PI+/PSA-), or injured plasma membrane and acrosome (PI+/PSA+). The spermatozoa were also classified as carriers of high, medium, and low mitochondrial potential (ΔΨ), according to previously described classification (Castro et al., 2016).

### Statistical analysis

The statistical analysis was carried out taking into account the completely randomized experimental design with three treatments (diets) and repeated measures over time (eight collections). Initially, the response variables were tested for the assumptions of error normality and variance homogeneity by Kolmogorov-Smirnov test. The data on the variables with distribution outside normality were transformed through the log function. The data obtained from the variables assessed both in *in natura* semen and in cryopreserved semen were submitted to analysis of variance using the procedure PROC GLM of the software SAS. This model considered the effects of the lipid source (3 classes; CONT, PALM, PODD), of the semen processing (2 classes; *in natura* or cryopreserved), of the time of collections (8 classes; collections 1 to 8), and of the interaction among the factors. The variables assessed only prior to or after cryopreservation were submitted to analysis of variance to test only the effect of lipid source, of the time of collections, and of the interaction between these factors. Since the effect of the time of collections and the interactions involving this factor were not significant in any situation, the interactions were removed from the models and values represent the averages of the whole period. The data were submitted to a comparison of means by Tukey’s test. All tests adopted a significance level of 5%. The analyses were performed using the software package SAS version 9.3 (SAS, 2011).

## Results

Daily dry matter intake in relation to live weight was not impacted by lipid supplementation. No significant difference in intake was observed among the CONT (1.4±0.1%), PALM (1.1±0.2%), or PODD (1.1±0.1%) diets.

The lipid profile of seminal plasma (Table 4) indicated lower proportions of caproic (C6:0; P=0.0028), pelargonic (C9:0; P=0.0065), and hendecanoic (C11:0; P=0.0001) acids among the bulls receiving lipid supplementation. The proportion of palmitic acid (C16:0; P=0.0394) was the highest in the PALM diet, whereas the highest percentage of tricosanoic acid (C23:0; P=0.0179) was found for the PODD diet. Linoleic acid (C18:2; P=0.05) was the only unsaturated fatty acid found at higher levels in the seminal plasma of bulls in the PALM group compared to control ones. Linolenic acid (C18:3), an unsaturated acid absent from the seminal plasma of control bulls, was detected in the PALM and PODD groups with no significant difference between concentrations.

**Table 4:** Fatty acid composition (mean±standard deviation) of seminal plasma of buffalo bulls supplemented or not with dietary lipids.

**Fatty acid (%)**	**CONT (n=32)**	**PALM (n=32)**	**PODD^†^ (n=24)^‡^**
Butyric acid (C4:0)	-	0.1±0.1	-
Caproic acid (C6:0)	2.7±1.9ª	1.4±1.4^b^	1.2±1.7^b^
Caprylic acid (C8:0)	0.1±0.5	-	0.1±0.1
Pelargonic acid (C9:0)	10.0±7.2ª	4.5±5.7^b^	5.3±7.2^b^
Capric acid (C10:0)	0.1±0.3	-	-
Undecanoic acid (C11:0)	4.9±3.1ª	2.0±2.1^b^	1.7±2.3^b^
Lauric acid (C12:0)	-	-	-
Tridecanoic acid (C13:0)	-	-	-
Myristic acid (C14:0)	0.1±0.7	0.3±0.8	-
Palmitic acid (C16:0)	3.7±2.6^b^	9.8±14.2ª	6.8±7.8^ab^
Margaric acid (C17:0)	0.4±1.3	1.1±4.0	0.2±0.8
Stearic acid (C18:0)	2.5±2.0	2.7±2.6	2.7±4.9
Arachidic acid (C20:0)	-	-	0.1±0.3
Behenic acid (C22:0)	-	-	-
Tridecanoic acid (C23:0)	1.7±2.0^b^	1.7±2.4^b^	5.3±8.6ª
Lignoceric acid (C24:0)	0.2±0.7	0.4±0.8	0.2±0.8
Palmitoleic acid (C16:1)	-	-	-
Oleic acid (C18:1)	5.2±2.5	8.7±13.1	6.5±7.5
Linoleic acid (C18:2)	1.1±2.6^b^	4.8±8.1ª	2.8±5.4^ab^
Linolenic acid (C18:3)	-	0.2±0.8	0.1±0.2
Erucic acid (C22:1)	1.9±2.6	2.9±3.5	4.4±7.0

a,b,c Means with different superscript letters on the same row are significantly different (P<0.05). †CONT = control group; PALM = crude palm oil group; PODD = palm oil deodorization distillate group; ‡ n=number of ejaculates.

The lipid composition of the sperm membrane (Table 5) indicated a reduction in the proportion of hendecanoic acid (C11:0; P=0.0179) among the PODD animals. The percentage of myristic acid (C14:0) did not differ between controls and the supplemented groups, but its values in PALM bulls were higher than in those in the PODD group (P<0.05). Palmitic acid (C16:0) did not differ between PALM and PODD, but the values of the former were higher than those of controls (P<0.05). Tridecanoic (C13:0), arachidic (20:0), and docosanoic acid (22:0) were detected only in PALM bulls. Lignoceric acid (C24:0; P=0.0162) was present at higher amounts in PALM and PODD compared with CONT. No difference was found in the concentration of unsaturated fatty acids in the sperm membrane between groups. Linoleic acid (C18:3) was detected in the PALM group, but not in CONT or PODD.

**Table 5:** Fatty acid composition (mean±standard deviation) of plasma membrane of spermatozoa from buffalo bulls supplemented or not with dietary lipids.

**Fatty acid (%)**	**CONT (n=32)**	**PALM (n=32)**	**PODD^†^ (n=24)^‡^**
Butyric acid (C4:0)	-	-	-
Caproic acid (C6:0)	2.1±1.0	1.4±1.2	1.3±1.2
Caprylic acid (C8:0)	0.3±0.8	-	0.3±0.6
Pelargonic acid (C9:0)	8.7±7.9	6.1±5.8	5.4±5.0
Capric acid (C10:0)	0.4±0.8	-	0.1±0.5
Undecanoic acid (C11:0)	3.4±2.5ª	2.2±1.7^ab^	2.0±1.9^b^
Lauric acid (C12:0)	-	-	0.1±0.5
Tridecanoic acid (C13:0)	-	0.1±0.1	-
Myristic acid (C14:0)	0.7±1.1^ab^	1.2±2.5ª	0.1±0.4^b^
Palmitic acid (C16:0)	5.4±1.7^b^	10.8±9.0^a^	7.2±8.0^ab^
Margaric acid (C17:0)	0.3±1.2	1.7±4.6	0.7±1.2
Stearic acid (C18:0)	2.5±1.1	3.9±3.1	3.9±5.1
Arachidic acid (C20:0)	-	0.1±0.1	-
Behenic acid (C22:0)	-	0.1±0.1	-
Tridecanoic acid (C23:0)	2.3±2.6	1.8±2.6	3.5±5.1
Lignoceric acid (C24:0)	1.9±3.5^b^	4.0±5.1ª	4.2±3.6ª
Palmitoleic acid (C16:1)	0.1±0.4	1.4±7.0	0.7±2.0
Oleic acid (C18:1)	4.3±1.2	7.1±7.6	7.6±9.6
Linoleic acid (C18:2)	1.1±1.1	2.5±2.1	1.9±3.9
Linolenic acid (C18:3)	-	0.1±0.1	-
Erucic acid (C22:1)	2.0±2.3	2.0±2.2	3.2±4.7

a,b,c Means with different superscript letters on the same row are significantly different (P<0.05). †CONT = control group; PALM = crude palm oil group; PODD = palm oil deodorization distillate group; ‡ n=number of ejaculates.

Progressive motility did not differ among the treatments for *in natura semen*. The cryopreservation process reduced progressive motility in all experimental groups. However, the progressive motility in cryopreserved semen was higher for the bulls that received lipid supplementation (PALM and PODD) (Table 6). Sperm vigor reduced after cryopreservation for CONT animals (P<0.0001), but remained similar to the original condition in those in the PALM and PODD groups.

**Table 6:** Sperm characteristics of *in natura* and cryopreserved semen (mean±standard deviation) of buffalo bulls fed diets enriched with lipids from crude palm oil (PALM) or from palm oil deodorization distillate (PODD).

**Variable**	**CONT (n=32)**	**PALM (n=32)**	**PODD (n=24)^‡^**	**CV^†^ (%)**
Progressive motility (%)				
*In natura*	70.5±1.2^A^	72.7±1.4^A^	70.7±1.2^A^	7.4
Cryopreserved	60.2±1.2^Bb^	67.9±1.4^Ba^	65.2±1.3^Ba^	7.8
Vigor (%)				
*In natura*	3.2±0.1^A^	3.2±0.1	3.2±0.1	0.5
Cryopreserved	2.5±0.1^B^	3.3±0.1	2.9±0.1	0.5
Viability (%)				
*In natura*	70.9±1.4	72.8±1.7	69.2±1.4	10.7
Cryopreserved	61.1±1.3^b^	69.4±1.5^a^	67.8±1.3^a^	9.2
Major defects (%)				
*In natura*	12.2±0.8^a^	9.3±1.0^b^	13.2±0.8^a^	6.0
Cryopreserved	12.4±0.8^a^	9.4±1.0^b^	11.2±0.8^ab^	5.1
Minor defects (%)				
*In natura*	3.7±0.5	3.0±0.5	2.7±0.5	3.5
Cryopreserved	3.3±0.5	3.5±0.5	2.7±0.5	2.7
Total defects (%)				
*In natura*	16.0±1.0^a^	12.3±1.2^b^	16.0±1.0^a^	7.3
Cryopreserved	15.8±1.0	12.9±1.2	14.0±1.0	5.9

a,b,c Means with different small superscript letters on the same row are significantly different (P<0.05); ^A,B^ Means with different capital superscript letters on the same column are significantly different (P<0.05). † CV = coefficient of variation; ‡ n=number of ejaculates.

Sperm viability of *in natura* semen did not differ among the groups. However, a significant positive effect of lipid supplementation on cryopreserved semen was observed, evidenced by the higher percentage of live spermatozoa in the animals in the PALM and PODD groups (P<0.0001). The bulls in the PALM group had a lower percentage of major sperm defects both in *in natura* and cryopreserved semen (P<0.0001). No significant difference was found in the occurrence of minor sperm defects both before and after cryopreservation. However, bulls in the PALM group had a lower percentage of total sperm defects in *in natura* semen (P<0.0001).

No significant difference was seen in the percentage of spermatozoa with intact plasma membrane and acrosome (PI-/PSA-; CONT=40.1% vs PALM=44.2% vs PODD=43.9%; P>0.05) (Table 7). Neither was a significant effect seen for the cells with injured acrosome regardless of the plasma membrane being intact (PI-/PSA+; CONT=23.9% vs PALM=17.9% vs PODD=23.5%; P>0.05) or not (PI+/PSA+; CONT=21.8% vs PALM=20.4% vs PODD=20.1%; P>0.05). A significant difference was seen in the percentage of spermatozoa with injured plasma membrane and intact acrosome (PI+/PSA-) between groups PALM and PODD (CONT=13.9% vs PALM=17.2% vs PODD=12.2%; P=0.034). The population of spermatozoa with high ΔΨ increased (CONT=25.6% vs PALM=31.5% vs PODD=32.0%; P=0.008) while the percentage of cells with medium ΔΨ decreased (CONT=35.0% vs PALM=24.3% vs PODD=26.9%; P=0.002) in the semen of animals receiving lipid supplementation. No significant difference was seen in the percentage of spermatozoa with low ΔΨ (CONT=39.3% vs PALM=43.3% vs PODD=40.2%; P>0.05).

**Table 7:** Plasma membrane and acrosome integrities (%) and mitochondrial potential (%) of cryopreserved spermatozoa from buffalo bulls fed diets enriched with lipids from crude palm oil (PALM) or from palm oil deodorization distillate (PODD).

**Variable**	**CONT (n=32)**	**PALM (n=32)**	**PODD (n=24)**
Plasma membrane and acrosome integrity (%)^†^			
PI-/PSA-	40.1±2.3	44.2±2.8	43.9±2.4
PI-/PSA+	23.9±2.0	17.9±2.4	23.5±2.1
PI+/PSA-	13.9±1.4 ^ab^	17.2±1.7^a^	12.2±1.4^b^
PI+/PSA+	21.8±1.5	20.4±1.8	20.1±1.5
Mitochondrial potential (%)			
High ΔΨ	25.6±1.6^b^	31.5±1.9^a^	32.0±1.6^a^
Medium ΔΨ	35.0±2.1^a^	24.3±2.5^b^	26.9±2.1^b^
Low ΔΨ	39.3±2.2	43.3±2.6	40.2±2.3

a,b,c Means with different superscript letters on the same row are significantly different (P<0.05). † PI-/PSA-, intact plasma membrane and acrosome; PI-/PSA+, intact plasma membrane and injured acrosome; PI+/PSA-, injured plasma membrane and intact acrosome; PI+/PSA+, injured plasma membrane and acrosome. ΔΨ, mitochondrial potential. ‡ n=number of ejaculates.

## Discussion

One of the most sensitive aspects of supplementing ruminants with fats lies on the fact that the gradual increase in the proportion of dietary lipids determines a linear reduction in daily dry matter intake (Costa et al., 2011; Berchielli et al., 2012). However, in the present study, adding 3% fatty acids to dry matter did not negatively impact feed intake, which was virtually identical in all groups. That was likely because the level of lipid use was below the minimum concentration able to cause intake reduction. Some evidence indicates that intake is reduced only by adding lipid at over 7% of dry matter, which results in lower ruminal degradability (Valinote et al., 2006).

However, the distinction in chemical composition of the diets resulted in different lipid intake among the groups. The seminal plasma of supplemented animals comprised fewer short-chain fatty acids such as C6:0, C9:0, and C11:0. Also, the C16:0 increased more in the seminal plasma of supplemented animals with crude palm oil than those fed with deodorization distillate, in spite of the high concentrations of this longer-chain fatty acid in both supplements (37.97 and 46.05%, respectively). The reason for this contradictory result may be the fact that the response to fat acids or even other nutritional components supplementation is not supposed to be linear. This phenomenon has been observed in many experiments testing nutraceutical effects of different fatty acid sources and/or concentrations of the same lipidic compound to feed rabbits (Mourvaki et al., 2010), roosters (Bongalhardo et al., 2009), fishes (Pustowka et al., 2000), and bovine (Gürler et al., 2015), among other species. This result is particularly relevant because the fatty acids present in seminal plasma are important for protecting spermatozoa since they take part in antioxidative processes during ejaculation and sperm capacitation (Kelso et al., 1997).

High C18:2 values in the seminal plasma of the animals supplemented with crude palm oil are also explained by the higher concentration of this fatty acid in the diet (9.52%). C18:2 is important because it is converted into C20:4, which, in turn, is directly involved in sperm membrane physiology (Wathes et al., 2007). In addition, lower quality of bovine cryopreserved semen has been reported as a consequence of lower C18:2 and C20:4 concentrations in spermatozoa (Argov et al., 2007). The difference in C18:2 abundance in the biological matrices analyzed in the present study associate with previous results obtained by our research group in complementary experiments with water buffaloes (Gonçalves et al., 2014; Santos et al., 2017) allow inferring that seminal plasma composition varies more immediately than sperm membrane composition due to the lipemic effect noted when animals are fed diets rich in fats (Silva et al., 2014).

Overall, the prevailing fatty acids in spermatozoa were C9:0, C16:0, and C18:1, identified in this order of proportion in the animals of the control group. Those fatty acids maintained their importance in the sperm membrane composition of the treated animals and only their preponderance changed, which was expressed by higher percentages of C16:0 and C18:1 in the bulls supplemented with crude palm oil and deodorization distillate, respectively. Compared with the control group, the higher concentration of C14:0 and C16:0 in the sperm membrane of the animals supplemented with palm oil indicated that the amount of these fatty acids in the supplement was sufficient for them to be ingested and secreted into the seminal plasma at a high enough concentration for it to be incorporated into the sperm membrane. In *in vitro* experiments, sperm membranes start to incorporate exogenous C16:0 very rapidly, in an interval as short as two minutes after incubation (Neill and Masters, 1971). C24:0 concentration was higher in the animals fed additional lipids, although this fatty acid was not identified in the chemical analysis of the supplements.

For its part, C18:3 is an essential fatty acid that is converted into active metabolites such as C20:5 and C22:6 (Gulliver et al., 2012) and protects the structural and functional integrity of buffalo spermatozoa during cryopreservation (Ejaz et al., 2017). Although C18:3 was detected in the plasma membrane of spermatozoa from bulls supplemented with crude palm oil, their derived metabolites were not detected. Dietary supplementation with unsaturated fatty acids reportedly increases the quality of cryopreserved buffalo semen (Gonçalves et al., 2014) likely because they modulate the structure and lipid composition of cell membranes (Fan et al., 2003; Calder, 2012). However, in the present study, the most significant changes in sperm membrane composition were effectively related to the differences in expression of saturated fatty acids.

It is known that cryopreservation may impair spermatozoa functionality due to irreversible damage that compromises the integrity of the sperm membrane, acrosome, and mitochondria, which affects cell motility (Minervini et al., 2013; Kumar et al., 2014; Celeghini et al., 2008). Such damages have been credited to the reduction in unsaturated fatty acids and to the increase in saturated fatty acids occurring during cryopreservation. Furthermore, cryopreservation reduces the content of phospholipids such as sphingomyelin, a structural component of the sperm membrane (Flesch and Gadella, 2000). Thus, more relevance has been ascribed to the role of unsaturated fatty acids in the cryopreservation process. Moallem et al. (2015) have reported higher progressive motility after cryopreservation in bovine bulls supplemented with linolenic acid (43.23%) compared with a source of saturated fatty acids (38.66%). Likewise, supplementation with polyunsaturated fatty acids proved useful to increase sperm motility in rams (Jafaroghli et al., 2014). Nonetheless, in the present study, the increase in progressive motility and maintenance of vigor after cryopreservation followed the increase in saturated fatty acids concentration in the sperm membrane of the animals supplemented with crude palm oil (C14:0, C16:0, and C24:0) or with deodorization distillate (C24:0). Since viable sperm population, represented by non-eosinophilic cells in supravital staining, is significantly and positively correlated with progressive motility (Mandal et al., 2003), our results suggest saturated fatty acids also play an important role in maintaining the structural integrity of sperm membranes, with a positive effect on sperm functionality, and that this effect is being underestimated, at least for buffalo spermatozoa.

The higher sperm viability observed in thawed semen of animals supplemented with lipids supports the evidence that the population of live cells after cryopreservation is directly related to the lipid composition of the sperm membrane (Hammerstedt, 1993). An increase in sperm viability has been reported when buffalo bulls were supplemented with C18:3 (Tran et al., 2016). The current study reveals that feeding lipid supplementation added with 3% crude palm oil, which contained higher ratio of unsaturated/saturated fatty acids and higher C16:0 values, favored sperm morphology normality and reduced the percentage of spermatozoa with major and total defects in the ejaculates. The same positive effect was recently described for taurine bulls fed dietary supplementation based on a mix of linseed oil and palm oil, which significantly increased membrane integrity (10-15%), and decreased sperm abnormalities (5-9%) after freezing-thawing (Khoshniat et al., 2020). In the current study, besides reducing the incidence of major morphological damage that could be caused to cells by cryopreservation, crude palm oil had additional advantages as it improved sperm morphology (~4%) in raw semen.

It has been reported that the percentage of buffalo spermatozoa with intact sperm membrane may increase after supplementation with saturated (Santos et al., 2014) or unsaturated (Tran et al., 2016) fatty acids. However, in the present study, the similar incidence of intact plasma membrane cells and acrosome (PI-/PSA-) among the groups showed that adding 3% saturated and unsaturated fatty acids to the diets was not sufficient to change this characteristic. Since cryopreservation impairs sperm membrane and acrosome integrity (Celeghini et al., 2008), and as these are essential requirements for fertilizing, a higher level of dietary lipids might be needed to change the subpopulation of PI-/PSA- cells.

As mitochondrial potential is a characteristic directly related to energy production, it may impact sperm motility (Flesch and Gadella, 2000) For this reason, one of the most applicable results of the current study is associated with different sperm subpopulations regarding their mitochondrial potential. Membranes integrity is crucial for mitochondrial function and depends on the supply of proteins and phospholipids (Schenkel and Bakovic, 2014). Mitochondrial membranes are composed by low levels of sphingolipids and cholesterol, and higher amounts of phosphatidylcholine, phosphatidylethanolamine, and cardiolipin, comprising 40, 30, and 15% of total mitochondrial phospholipids, respectively (Osman et al., 2011). Rupture of the external mitochondrial membrane or the mitochondrial membrane permeabilization cause the leakage of proteins of the intermembrane space, which leads to cell death (Sesso et al., 2012). Some of the lipids present in the mitochondrial membranes have to be imported, at least in the form of precursors (Monteiro et al., 2014). Considering the mitochondrial biogenesis and the anatomic peculiarity of sperm mitochondrial sheath, mitochondria might also benefit from changes in lipaemic profile (Silva et al., 2014) and higher availability of fatty acids to increase their structural resilience during spermatogenesis. As much of the cytoplasmic mitochondria is lost during spermiogenesis, the morphological changes in 72-80 remaining mitochondria of late spermatids and sperm appear to prepare them for structurally fitting in sperm flagellum and arrange around the developing spermatid tail (Vertika et al., 2020). Therefore, dietary fatty acids, as well as disturbance of enzymes involved in phospholipid remodeling, may have changed the phospholipid composition of the mitochondrial membranes (Monteiro et al. 2013), and altered the mitochondrial bioenergetics and sperm function, as previously reported (Durairajanayagam et al., 2020).

The increase in cell subpopulations with high mitochondrial potential, as observed in the animals supplemented with lipids, shows a positive effect of supplementation with unsaturated and saturated fatty acids on the function of sperm mitochondria. It is suggested that this effect may also derive from the composition of seminal plasma, whose short-chain fatty acid content is reduced, associated with an increase in long-chain fatty acids (C16:0 and C23:0, respectively, for the groups supplemented with crude palm oil or deodorization distillate) and with the higher C23:0 concentration (only in the animals that consumed palm oil). In spite of glycolysis being generally considered as the primary pathway for ATP production in mammalian sperm, spermatozoa of some species could switch the energy metabolic route to mitochondrial β-oxidative phosphorylation due to a complex system to use different substrates as fatty acids. Recent results suggest that sperm cells of boars utilize oleic acid and palmitic acid as substrates for energy generation (Zhu et al., 2020). Since the semen of supplemented animals had higher progressive motility than that of the control animals after cryopreservation, this event seems to be interlinked with the change in seminal plasma composition and with the increase in mitochondrial electric potential generation, energy production and sperm motility, also in water buffaloes.

Taken together, our findings provide evidence for the hypothesis that supplementing buffalo bulls with lipids enhances their semen quality. Nonetheless, there are also many interesting, biologically relevant sources of variation that require further investigation, such as the pathways for the synthesis of phospholipids of acrosomal and mitochondrial membranes resulting from the dietary fatty acids supplementation. To the extent of our knowledge, this is the first study showing the effect of diets enriched with lipids by describing that saturated and unsaturated fatty acids may together play an important role in maintaining the structural integrity and functionality of buffalo spermatozoa, culminating with higher semen quality.

## Conclusions

Dietary supplementation of buffalo bulls with crude palm oil increased the C18:2 profile in seminal plasma. Lipid supplementation increased the expression of saturated fatty acids in sperm membrane and enhanced important semen parameters such as progressive motility, sperm viability, and mitochondrial potential of cryopreserved semen, which are directly related to fertilization capacity. Crude palm oil had an additional advantage by improving sperm morphology in *in natura* semen and after cryopreservation. Therefore, these lipid sources from the palm oil industry, which are rich in saturated and unsaturated fatty acids, can be safely used in buffalo bull diets with a positive expectation of increased fertility of cryopreserved semen.
